# Postmortem Digital Image Correlation and Finite Element Modeling Demonstrate Posterior Scleral Deformations during Optic Nerve Adduction Tethering

**DOI:** 10.3390/bioengineering11050452

**Published:** 2024-05-02

**Authors:** Seongjin Lim, Changzoo Kim, Somaye Jafari, Joseph Park, Stephanie S. Garcia, Joseph L. Demer

**Affiliations:** 1 Department of Ophthalmology, Stein Eye Institute, Los Angeles, CA 90095, USA; seongjinlim@uabmc.edu (S.L.); sjafari@mednet.ucla.edu (S.J.); blueash1989@gmail.com (J.P.); teppie.garcia@gmail.com (S.S.G.); 2 Department of Ophthalmology, Kosin University, Busan 49267, Republic of Korea; czkim@kosin.ac.kr; 3 Neuroscience Interdepartmental Program, University of California, Los Angeles, CA 90095, USA; 4 Department of Neurology, University of California, Los Angeles, CA 90095, USA; 5 Department of Bioengineering, University of California, Los Angeles, CA 90095, USA

**Keywords:** biomechanics, digital image correlation, eye movement, finite element modeling, sclera

## Abstract

Postmortem human eyes were subjected to optic nerve (ON) traction in adduction and elevated intraocular pressure (IOP) to investigate scleral surface deformations. We incrementally adducted 11 eyes (age 74.1 ± 9.3 years, standard deviation) from 26° to 32° under normal IOP, during imaging of the posterior globe, for analysis by three-dimensional digital image correlation (3D-DIC). In the same eyes, we performed uniaxial tensile testing in multiple regions of the sclera, ON, and ON sheath. Based on individual measurements, we analyzed eye-specific finite element models (FEMs) simulating adduction and IOP loading. Analysis of 3D-DIC showed that the nasal sclera up to 1 mm from the sheath border was significantly compressed during adduction. IOP elevation from 15 to 30 mmHg induced strains less than did adduction. Tensile testing demonstrated ON sheath stiffening above 3.4% strain, which was incorporated in FEMs of adduction tethering that was quantitatively consistent with changes in scleral deformation from 3D-DIC. Simulated IOP elevation to 30 mmHg did not induce scleral surface strains outside the ON sheath. ON tethering in incremental adduction from 26° to 32° compressed the nasal and stretched the temporal sclera adjacent to the ON sheath, more so than IOP elevation. The effect of ON tethering is influenced by strain stiffening of the ON sheath.

## 1. Introduction

The optic nerve (ON) faces a unique mechanical situation: at its anterior end, the ON is attached to the globe and undergoes large and numerous rotational perturbations, including more than 180,000 saccades daily that encompass a horizontal range of up to ±55° [1]. But the opposite end of the ON is anchored rigidly to the bone surrounding the optic canal. Eye movements thus perturb the intraorbital ON, although until recently this phenomenon was underappreciated [2]. The length of the ON is usually insufficient to permit unhindered rotation, tethering the globe in adduction [2]. The stretched ON and its sheath were thus reasoned to induce traction on the optic disc and peripapillary region [2]. Sibony used optical coherence tomography (OCT) to demonstrate deformations of the peripapillary Bruch’s membrane associated with horizontal duction [3]. Further, OCT studies quantified relative displacements [4], tilting [5], local area changes [6], and shear strains [7] in the optic disc during horizontal duction. In addition to horizontal duction-related anteroposterior deformations, in-plane deformations within the retina have also been investigated using confocal scanning laser ophthalmoscopy (cSLO) [8,9]. Angiographic OCT has demonstrated that adduction is associated with disc and vascular strains much larger than those reported for IOP elevation and pulsatile perfusion, and adduction also causes optic disc compression and an increase in peripapillary vascular volume [10]. Magnetic resonance imaging (MRI) also demonstrated in normal subjects that ON strain during adduction is due to uniform stretching along its length [11].

Finite element models (FEMs) have been used to elucidate the effects of mechanical loading on the eye, including intraocular pressure (IOP) elevation [12,13] and eye rotation [14]. Wang et al. [15,16] and Shin et al. [17] developed FEMs of disc deformations during ductions. The sensitivity analysis suggested that unfavorable combinations of properties would concentrate adduction-related stresses at the sites most commonly affected by primary open-angle glaucoma (POAG) [18]. However, because stresses and strains from adduction are dependent on relative magnitudes of stiffness for different ocular regions [18], the linkage between the adduction tethering and ocular deformations is not necessarily straightforward. If the ON and its sheath are much more compliant than the sclera, the taut ON might not transmit sufficient force to significantly deform the eyeball. Since the ON sheath is known to exhibit a low elastic modulus in the small strain regime [19], additional information is required to quantitatively estimate the effects of ON traction on the globe.

Three-dimensional digital image correlation (3D-DIC) of the eye in response to inflation loading under varying levels of IOP has been used to estimate parameters of constitutive equations to characterize mechanical properties of the posterior sclera [20]. Another approach to characterization has employed a 3D digitizer and laser speckle interferometry during inflation to characterize the mechanical properties of the posterior sclera [21]. Because regions of interest for both studies were limited to the posterior sclera, the ON and the anterior globe were removed for inflation experiments [20,21]. The current study was performed to further investigate potentials for adduction tethering to load the posterior human sclera. Extensive in vivo MRI [22,23,24] and posterior intraocular imaging [5,8,9,10,11,25,26] data exist demonstrating effects of incremental adduction from the average threshold of ON tethering at 26°, by an additional 6° to 32° adduction, a practical experimental maximum for humans. These imaging studies provide high resolution of a limited area of internal ocular structures, and for MRI low resolution of the entire globe and a wide range of orbital structures. What has heretofore been missing has been high-resolution imaging of the external surface of the posterior sclera. Since high-resolution imaging of the external surface of the posterior sclera is impossible in vivo [27], we designed an ex vivo experiment using 3D-DIC [28] to determine how adduction replicating 26–32° adduction, previously studied by in vivo MRI [23,24], deforms the posterior sclera. Furthermore, we aimed to characterize mechanical properties for each relevant region of each specimen after the 3D-DIC experiment. Based on individual eye anatomy and local tissue material properties, we sought to compare 3D-DIC findings during adduction and IOP elevation with individualized FEMs of the same conditions. Such comparisons represent a strong test of the proposition that adduction tethering may significantly load the posterior eye and allow comparison of its effect relative to IOP.

## 2. Materials and Methods

### 2.1. Overall Workflow (Figure 1)

Human eyes with attached ONs averaging 17.9 ± 2.9 (standard deviation, SD) mm long were shipped to the laboratory by the Lions Gift of Sight Eye Bank (Saint Paul, MN, USA) shortly after the donors’ deaths. Each specimen was tested using 3D-DIC under loading by adduction tethering from 26–32°, as well as by IOP elevation. The globes and ONs were then divided into specimens that were subjected to tensile testing. Stress–strain curves obtained by tensile testing were employed for individual FEMs of eyes under IOP elevation and adduction. Additionally, these were compared with 3D-DIC results.

**Figure 1:**
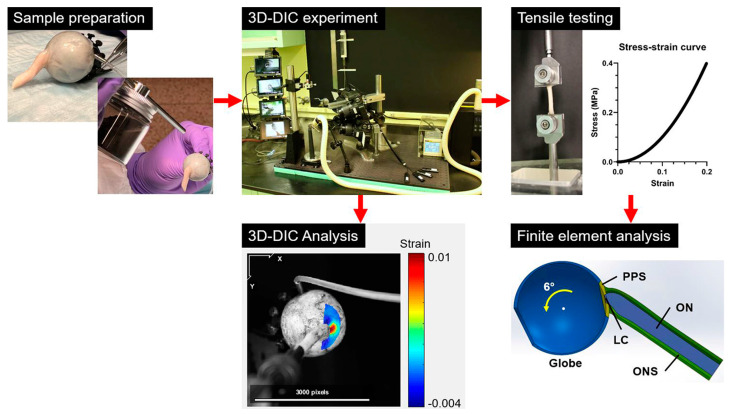
Workflow sequence indicated by red arrows. 3D-DIC was performed in whole globes to quantify effects of adduction tethering and IOP elevation. Uniaxial tensile testing of tissues from these globes was then conducted to characterize material properties of local regions of each specimen, and used to generate individualized FEMs that were compared with individualized 3D-DICs. Heat map from DIC shows strains along the horizontal direction in the image plane, superimposed on photo of specimen. PPS—peripapillary sclera. LC—lamina cribrosa. ON—optic nerve. ONS—optic nerve sheath.

### 2.2. Specimen Preparation

Specimen donors had consented to use of their eyes for research. Eyes were harvested and transported using procedures identical to those for corneal transplantation donation. Specimens wrapped in cotton gauze moistened with saline in insulated containers cooled with ice packs were air shipped to the laboratory an average of 39.1 ± 12.9 h following enucleation. Tensile viscoelastic properties of sclera are not altered by storage for 72 h [29]. Donors were white, with average age 74.1 ± 9.3 years. There were five males and two females.

**Figure 2:**
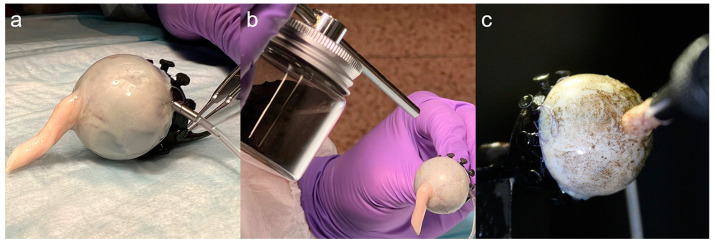
Sample preparation. (**a**) Globe was sutured to fixation ring, and infusion cannula inserted in anterior sclera over the pars plana. (**b**) Speckles of cast iron dust were randomly applied to the surface by using a pneumatic applicator. (**c**) Speckle pattern on the sclera permitted optical tracking of local scleral deformation.

After removal of any overlying tissues, 6-0 coated Vicryl suture (Ethicon, Raritan, NJ, USA) was used to attach the anterior sclera onto multiple suturing posts on a custom aluminum fixation ring, passing partial thickness through the perilimbal sclera without perforation. The 45° internally beveled ring had a 20 mm outer diameter and 16.5 mm inner diameter. Right eyes were sutured into the fixation ring with the superior region upward, whereas for left eyes, the orientation was downward. A stab incision was made in the anterior sclera over the pars plana 3.5 mm from the limbus using a 23-gauge ophthalmic micro-vitreoretinal blade (Alcon, Ft. Worth, TX, USA), through which a 23-gauge cannula (Bausch + Lomb, Laval, QC, Canada) was inserted to maintain IOP by Ringer’s lactate fluid infusion (Figure 2a). Then, cast iron dust of 150 mesh size (Chemical Store Inc., Clifton, NJ, USA) was randomly scattered on the surface of the eyeball by using a pneumatic applicator (Elaimei, Portsmouth, UK) (Figure 2b) to create an irregular pattern suitable for imaging to compute deformations by 3D-DIC (Figure 2c).

### 2.3. Apparatus for 3D-DIC

The infusion cannula was connected to a fluid column containing Ringer’s lactate solution (B. Braun Medical Inc., Bethlehem, PA, USA) via an infusion set (MedSource, Rancho Santa Margarita, CA, USA), allowing IOP control by changing the height of the infusion fluid column relative to the ocular center. The reference level of hydrostatic pressure was 15 mmHg and could be increased to 30 mmHg. During the experiment, warm mist at 30 °C from an ultrasonic humidifier (Asakuki, Rochester, NY, USA) was directed to the specimen to suppress dehydration. Because dissection of tissues leads to the shrinkage of the samples from whole organs, preconditioning is often performed to stretch crimped fibers within the samples [30]. Whole globes used in this experiment were not dissected, so this rationale for preconditioning is not applicable to the present study. Although Bianco et al. demonstrated an effect of preconditioning for numerous cycles of very high IOP loading of the whole tree shrew globe [31], inflation testing in bovine and porcine ocular tissues has demonstrated minimal effects of preconditioning [31,32]. Moreover, our prior tensile study of human posterior sclera, optic nerve, and optic nerve sheath strip specimens found only a modest quantitative effect of preconditioning up to 2% strain [33]. Therefore, preconditioning was not performed for the intact whole-eye 3D-DIC experiment.

A laser cross-alignment projector (OXLasers Co., Ltd., Shanghai, China) was mounted above the globe to align the eye ocular center with the rotational center of a 300 mm radius curved translational bearing (Misumi, Tokyo, Japan). The distal end of the ON was coaxially clamped by a custom gripper on a rod in a mechanical series with a force sensor LSB200 (FUTEK Advanced Sensor Technology, Irvine, CA, USA) that traveled along the bearing arc. The position of the ON clamp on the terminal end of the rod was vernier adjustable. The globe was positioned until its center corresponded to the projected laser cross center. Then, the initial rotational orientation of the eye was set by a precision rotation mount (Thorlabs, Newton, NJ, USA). Initial ON tension was set by extending the position of its terminal support rod until the force sensor demonstrated detectable tension. The length of the straight ON and the transverse diameter of the globe were used for determining initial and deformed configurations of the ON, as described below.

**Figure 3:**
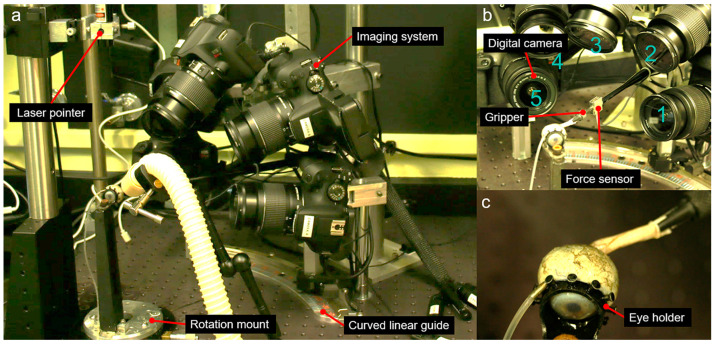
Setup for 3D-DIC. (**a**) Overall system, including the whole eye warmed and humidified via the white air tube, cameras, and curve guide for the gripper holding the distal ON. (**b**) Close-up of imaging system. (**c**) Magnified image of an eye in its ring holder, with the ON gripper at the upper left.

Five Canon EOS Rebel T7 digital single-lens reflex cameras (Canon, Tokyo, Japan) were fixed in multiple locations in manual focus mode (Figure 3). Each camera was fitted with an EF-S 18–55 mm lens (Canon, Tokyo, Japan), with focal length set to 55 mm, at which pincushion distortion was 0.2%. The cameras generated 6000 by 4000 pixel images, with each pixel approximately 14 µm square. Remote controllers RS60 E3 (Canon, Tokyo, Japan) were used to avoid camera vibration. Although conventional DIC can be performed with as few as two cameras [20], five cameras were employed because of occlusions of some ocular regions in some cameras due to the long ON and the coaxial gripper. Thus, four different pairs of cameras were utilized to capture images of the nasal, superior, and temporal regions of the posterior sclera (Figure 3b). Cameras 1 and 2 covered the nasal regions, while cameras 2, 3, and 4 captured images of the superior regions. Lastly, cameras 4 and 5 were dedicated to imaging the temporal regions.

### 2.4. Geometric Consideration for Adduction Loading

**Figure 4:**
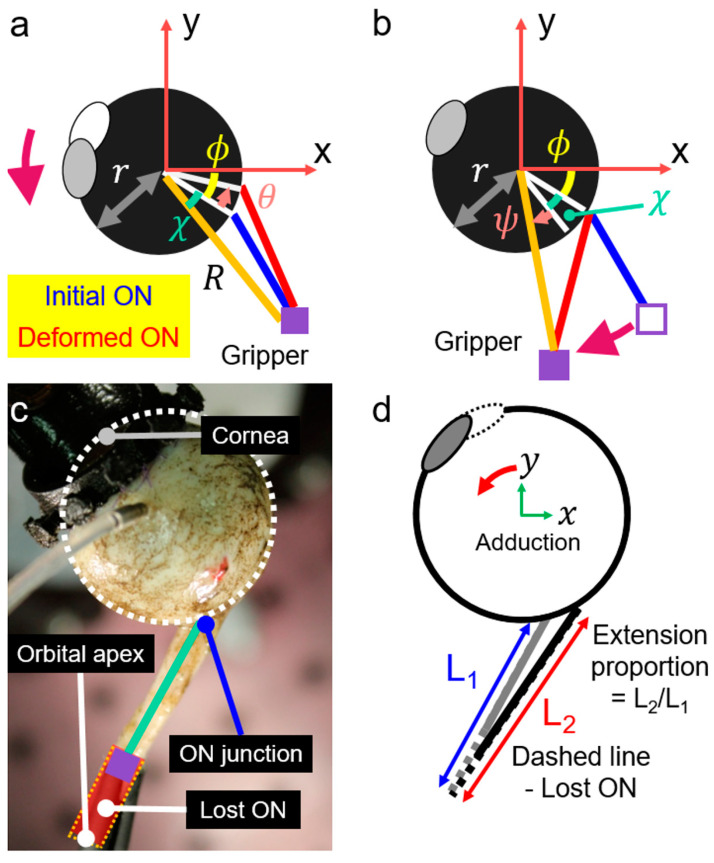
Schematic diagram for geometric computation. (**a**,**b**) Diagram demonstrating equivalence between rotation of eye versus shifting the ON gripper along its supporting arc. Colored arrow arcs indicate rotations. In these two panels, r is the globe radius and ϕ the azimuth of the ON junction. The gripper location can be specified by the distance R and the incremental angle χ, where θ indicates the angle of eye rotation and ψ the angular arc of travel for the gripper. The blue and red lines correspond to the ON path at the initial and deformed configuration. (**c**) Globe in eye holder with superimposed virtual orbital apex. Because of the offset between the globe center and the ON junction, the alignment of the ON and the clamp vary slightly from collinearity. (**d**) Extension proportion of the ON length during incremental adduction. Angles are exaggerated in panels b and d for diagrammatic clarity.

Because the ocular centroid coincided with the rotational center of the curved translational bearing, polar coordinates were convenient to specify the locations of the ON junction with the globe, and the terminal ON gripper. The initial configuration of the adduction angle was set to be 26° because this is the reported threshold for the high deformation regime in the optic disc. A kinematically equivalent approach was taken that maintained a fixed ocular center and cameras, but instead altered the relative rotational location of the ON support that constituted the virtual orbital apex. According to the nomenclature in Figure 4a,b, r represents the globe radius, and ϕ is the azimuth of the ON junction. At the same time, R represents the distance from the globe center to the gripper, and χ is the offset angle between the ON junction and the gripper. Whether rotating the eye, or moving the ON gripper along the arc support, the ON elongation caused by additional adduction to 32° can be computed from the law of cosines. When the gripper is rotated by ψ (Figure 4b), the ON length is (r^2^ + R^2^ − 2rRcos(χ + ψ))^1/2^. Because the ON length of the rotated eye is (r^2^ + R^2^ − 2rRcos(χ + θ))^1/2^, the condition θ = ψ also enforces equality of the ON lengths for both cases, since the triangles formed by the globe center, the gripper tip, and the ON junction are identical. This demonstrates that rotating the ON gripper is kinematically equivalent to rotating the eye.

Enucleation inevitably leaves behind a portion of the ON in the donor orbital apex, so a virtual orbital apex was employed (Figure 4c). Using average dimensions for healthy people [18], the distance between the globe center and the orbital apex was set at 41 mm, and the angle between a sagittal plane and a line from the globe center and the orbital apex was 22°. The angle between the fovea and the optic disc was assumed to be 17° [18]. The straight ON length was measured with a digital caliper. Then, the initial position of the gripper tip was adjusted by referring to the virtual orbital apex and the straight ON distance. The gripper tip was located along a line between the ON junction and the orbital apex, while the ON length was constant. At the deformed configuration, the distance between the orbital apex and the ON junction increases by adduction, and the extension proportion of the distance can be computed (Figure 4d). Hence, we stretched the remaining actual ON by the same proportion because the ON is straight under the conditions of this experiment.

### 2.5. Validation of 3D-DIC

**Figure 5:**
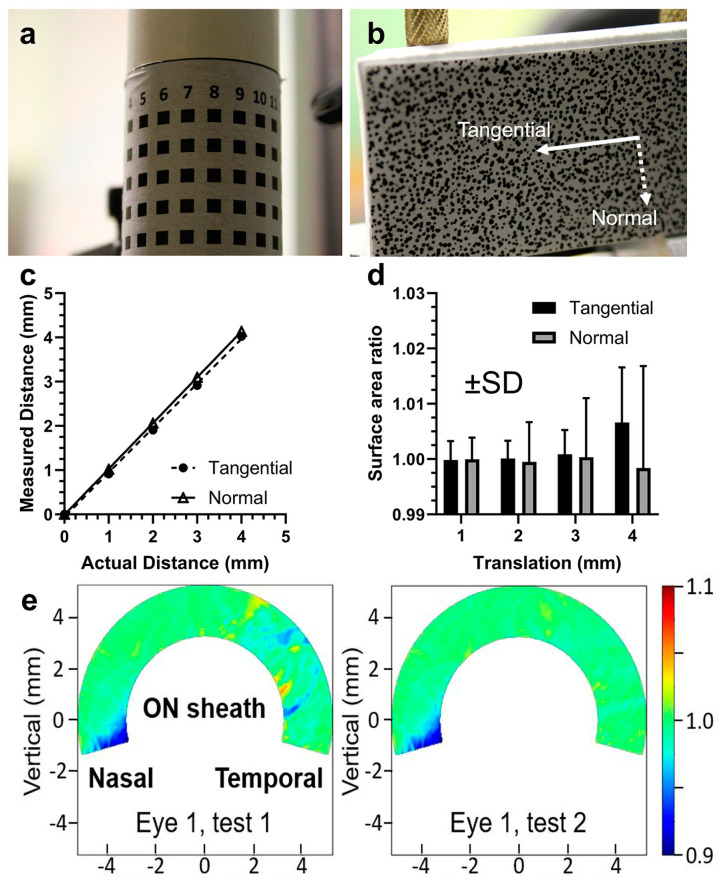
Verification systems for 3D-DIC. (**a**) Cylindrical standard to calibrate 3D coordinates. (**b**) Linear translation of pattern on vernier mount. (**c**) Measured displacement magnitudes by 3D-DIC correspond closely to actual. (**d**) Surface area ratios remained at nominal unity for normal and tangential translation up to at least 2 mm. (**e**) Repeatability of surface area testing of the same eye by 3D-DIC.

Images from each camera were cropped into 4000 by 4000-pixel squares. Calibration for determining 3D coordinates was conducted for each camera pair. The images were calibrated against known 3D coordinates of pattern features on a 25.4 mm diameter cylinder having dimensions similar to the human eye diameter (Figure 5a). Calibration was performed in manually selected overlapping regions for each camera pair. Then, image point coordinates were matched to known 3D coordinates to obtain direct linear transformation parameters [34] for each camera.

Verification of DIC displacements was performed using a standard consisting of a sheet of randomly patterned paper supported by two pillars (Figure 5b) mounted on a vernier stage that precisely controlled translation. Displacement magnitudes and surface area ratios were measured for normal and tangential translation (Figure 5c,d). If the operation of DIC is valid, ideal computed displacements should be veridical with pattern translation, while surface area ratios should be unchanged. These criteria were used for validation of DIC.

### 2.6. Measurements by 3D-DIC

Images from each camera were taken before and after specimen loading by adduction, and by IOP elevation. Regions of interest were manually selected by considering overlapped random speckle image regions of the sclera captured by camera pairs for DIC between the reference and deformed states. By referring to calibration parameters and images from different cameras, 3D surfaces were reconstructed by using open source 3D-DIC algorithms [28] from which 3D displacements around the ON junction were estimated. Although 3D strains have been studied in inflation testing [20], this metric is not applicable for adduction. Strains obtained from DIC compute only surface deformations by assuming negligible changes in scleral thickness, but compressive deformation during adduction loading invalidates the assumption. Instead of directly computing strains by DIC, since the reconstructed surfaces were tessellated by triangular faces, estimated displacements were used to compute surface areas of triangle meshes and quantify deformations under loading as relative changes in surface areas.

### 2.7. Tensile Testing

After DIC testing was completed, each whole specimen was sharply divided into anterior, equatorial, posterior, peripapillary sclera; ON; and ON sheath. Except for the ON, each region was dissected into rectangular strips that were used for uniaxial tensile testing [33]. The dissection protocol was the same as in the previous report, including the use of circular trephines to obtain peripapillary scleral specimens [33]. Averaged lengths and widths of the dissected strips were 8.43 ± 1.66 mm and 2.11 ± 0.36 mm, respectively. As elsewhere described in detail, the tensile load cell consisted of a linear motor (Ibex Engineering, Newbury Park, CA, USA) and a sensitive force sensor with ±0.1% error (FUTEK Advanced Sensor Technology, Irvine, CA, USA) in series with a rod supported by a frictionless air bearing within an environmental chamber maintained by thermocouple control at 37 °C over a water bath so that humidity approached 100% [35]. Dimensions of tissues were measured by digital calipers (Mitutoyo Co., Kawasaki, Japan). Using closed feedback control, tissues were elongated until the force sensor indicated 0.02 N as the preloading condition. Then, 6 cycles of 5% preconditioning were applied because stress–strain curves converge after 3 cycles of 5% stretching [33]. After the preconditioning, tensile elongation was performed at 0.07 mm/sec as force and length were sampled at 5 Hz until the specimen ruptured. Engineering stress–strain curves were then plotted for each tissue region.

### 2.8. Analysis of Tensile Results

Stress–strain curves were fitted by polynomial curves using the built-in function in MATLAB 2017b (MathWorks, Natick, MA, USA). The orders of polynomial equations were manually fitted between 3 and 5 to best fit the data, but otherwise have no particular physical meaning. Tangent moduli were computed by differentiating the polynomial equations. For comparison with previously reported data [33], tangent moduli at strain levels of 3% and 7% were computed. For ON sheath data, bilinear regression of stresses and strains was conducted after logarithmic transformation, minimizing total squared errors. The intersection point between the two lines was considered to be the end of the toe region, as done elsewhere [36].

### 2.9. Simulation by FEMs

Individualized tensile data were used for individualized FEMs of each specimen. An individualized geometric model was created using SolidWorks 2021 (Dassault Systèmes, Vélizy-Villacoublay, France) for each specimen, encompassing the whole globe and attached ON. For each specimen, measured axial length, transverse globe diameter, and external ON sheath radius at the ON junction were employed (Table 1), while the inner radius of the ON sheath was set to 2.2 mm. Local tissue regions were parameterized individually according to tensile data collected for the anterior, equatorial, posterior, peripapillary sclera; lamina cribrosa; ON; and ON sheath, assuming isotropic hyperelasticity. Reduced polynomial models were used, and the selected model orders were based on a prior study [14]. For the lamina cribrosa, the stress–strain curves were averaged between those of ON and peripapillary sclera, following the previous approach [14,18]. Then the models were imported into the Abaqus 2023 simulation environment (Dassault Systèmes, Vélizy-Villacoublay, France). A total of 540,196 ± 64,198 s order tetrahedral meshes were used for individual FEMs, ensuring mesh convergence [14,18]. The Poisson ratio of tissues was set to 0.495, assuming near incompressibility. As boundary conditions, the ON and its sheath were fixed at the orbital apex, and the perturbation was globe adduction from 26° to 32°. The eye was constrained to rotate about its center. To simulate pre-stretch, stress–strain curves for the ON and its sheath were translated by 5% toward higher values along the strain axis. Models with IOP elevation from 15 to 30 mmHg, but without incremental adduction, were also simulated. The coordinates and connectivity of nodes were exported to define triangles on the scleral surface whose areas were locally computed before and after the loading to quantify local surface area changes.

### 2.10. Statistical Analysis

Paired t-testing, one- and two-way ANOVA, and linear regression were performed by using GraphPad Prism 9 (GraphPad Software version 9, San Diego, CA, USA). Generalized estimating equation (GEE) was performed using IBM SPSS 25 (Armonk, NY, USA) for multivariate analysis.

## 3. Results

### 3.1. Reliability and Repeatability of DIC 

By imaging a uniform pattern on the surface of a cylinder of known size, we calibrated the computation of 3D coordinates from pairs of 2D images (Figure 5a). The mean squared error of 3D coordinates was about 10 µm. Assuming that errors for the 3D coordinates for each camera pair are independent and normally distributed, the mean squared error of computed displacements from the pairs would be 14.1 µm. Calibration was also performed by vernier translation of a random pattern in normal and tangential directions (Figure 5b–d) to compare displacement magnitudes and surface area ratios with ideal values (Figure 5c,d). Errors were not significant for translation up to 2 mm in both normal and tangential directions. Since all experimental displacements were smaller than 2 mm, measurements were considered reliable. We also measured duplicate adductions for the same eye, obtaining similar heat maps of the surface area ratios (Figure 5e). The average absolute difference between the dimensionless heat map values across the domain was 0.006.

### 3.2. Scleral Deformations around the ON Sheath during Adduction

**Figure 6:**
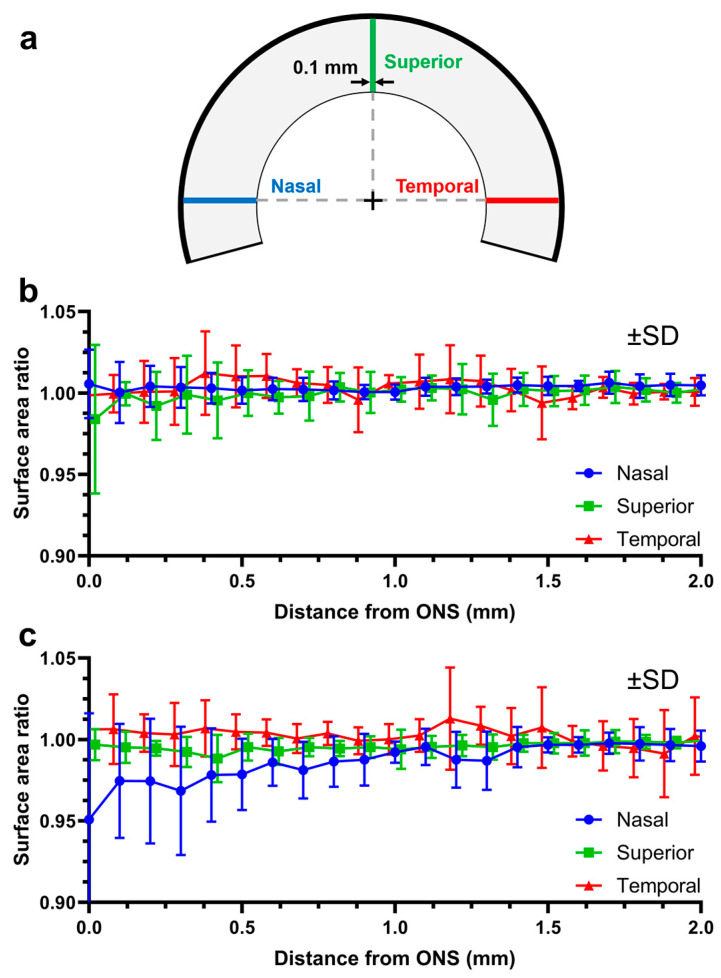
Propagation of changes in scleral strip surface area measured by DIC. (**a**) Three strips were analyzed. (**b**) IOP elevation from 15 to 30 mmHg. (**c**) Adduction by 6° changed surface area within 1.2 mm of the nasal edge of the ON sheath. Significance is by two-way ANOVA. SD—standard deviation.

After confirming the reliability and repeatability of 3D-DIC, we investigated whether scleral deformations occur under loading. Thus, three strips of 0.1 mm width over the scleral surface were defined along the nasal, superior and temporal directions (Figure 6a). Surface area ratios are less than one when regions are compressed and exceed one as the region expands. Scleral deformations were confined to within 2 mm from the margin of the ON sheath during both IOP elevation and adduction (Figure 6b,c).

**Figure 7:**
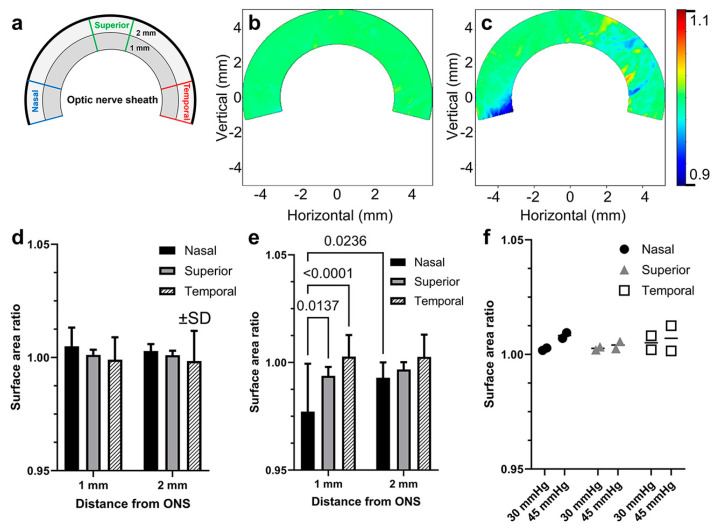
Relative changes in regional scleral surface area measured by DIC. (**a**) Diagram of scleral regions analyzed. Heat maps for the surface area ratio of a representative eye loaded by (**b**) IOP elevation from 15 to 30 mmHg and (**c**) adduction from 26° to 32°. Mean change in surface area ratios for all eyes during loading by (**d**) IOP elevation from 15 to 30 mmHg and (**e**) adduction from 26° to 32°. (**f**) Effect of moderate and extreme IOP elevation from 15 to 30 and 45 mmHg in scleral regions within 1 mm from the ON sheath (N = 2 eyes). Significance is by two-way ANOVA. SD—standard deviation.

Deformation was quantified by the change in local surface areas of the sclera, represented as ratios of loaded to unloaded areas in defined regions consisting of three fan-shaped sectors of a 30° central angle in the nasal, superior, and temporal regions, as diagrammed in Figure 7a. Because deformations occurred only within 2 mm from the ON sheath (Figure 6b,c), each sector was divided into two annular regions defined by radial distances of 1 and 2 mm from the ON sheath. Changes in surface area ratios were analyzed for each of the local areas defined in this way, during the elevation of the IOP from 15 to 30 mmHg, without altering the ON position (Figure 7d).

In the region 1 mm from the margin of the ON sheath, surface area ratios resulting from IOP elevation were 1.005 ± 0.008, 1.001 ± 0.002, and 0.999 ± 0.010 in nasal, superior, and temporal 1 mm regions (Figure 7d). In the region 2 mm from the margin of the ON sheath, the corresponding values were 1.003 ± 0.003, 1.001 ± 0.002, and 0.998 ± 0.013, respectively. Changes in surface area ratios caused by 6° incremental adduction were also computed (Figure 7e). In the region 1 mm from the margin of the ON sheath, the surface area ratios in the nasal region and superior regions were 0.977 ± 0.021 and 0.994 ± 0.004, and 1.003 ± 0.010 for the temporal region by adduction. The nasal region was compressed significantly within 1 mm of the ON sheath by adduction (*p* = 0.0236), but not at 2 mm. In the region 2 mm from the margin of the ON sheath, the corresponding values were 0.993 ± 0.007, 0.997 ± 0.003, and 1.003 ± 0.010, respectively. To ascertain that extreme IOP elevation affects the scleral surface, we examined the case of IOP elevation to 45 mmHg for two eyes (Figure 7f). Modest increases in surface area ratios in all nasal, superior, and temporal regions showed that the 3D-DIC analysis can detect small scleral deformations caused by extreme IOP elevation.

We quantitatively compared the effects of adduction and IOP loadings on various scleral regions by computing the mean absolute difference of surface area ratios from unity (Table 2). Adduction-induced appreciable deformations exceeded 2% within 1 mm nasal to the ON sheath during adduction tethering, while deformations were less than 1% elsewhere during adduction, as well as during IOP elevation from 15 to 30 mmHg.

### 3.3. Uniaxial Tensile Behavior

**Figure 8:**
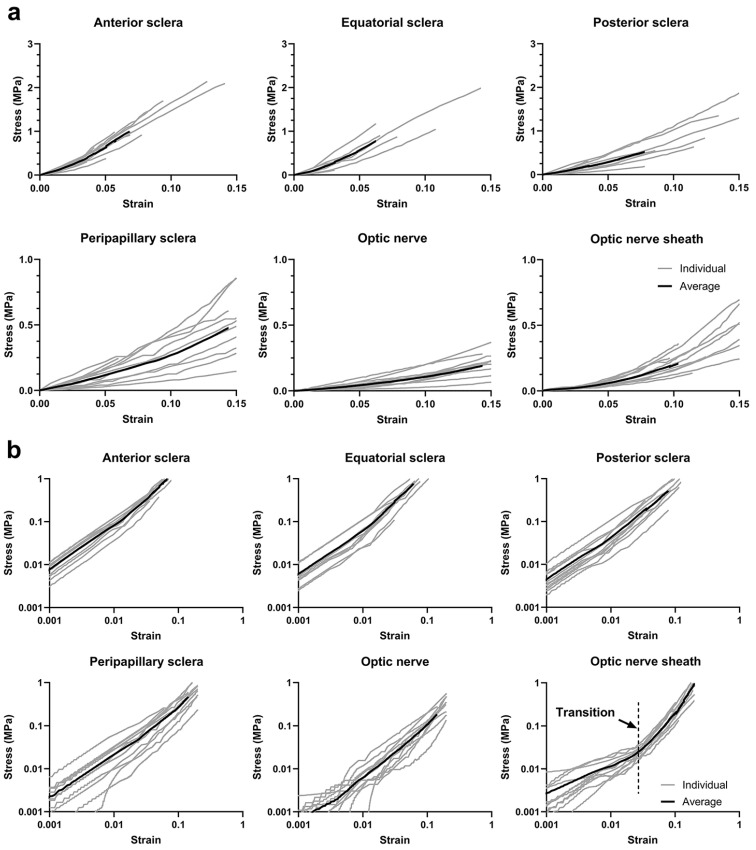
Individual (gray) and average (black) engineering tensile stress–strain curves for 11 human scleras, ONs, and ON sheaths: (**a**) linear scale and (**b**) log scale. Note that the ordinate range is larger in the top than lower rows.

Uniaxial tensile loading generated stress–strain curves from which tangent moduli were computed (Figure 8a). At 3% strains, tangent moduli of anterior, equatorial, posterior, and peripapillary sclera were 14.0 ± 3.4 MPa, 11.7 ± 3.5 MPa, 7.0 ± 2.9 MPa, and 2.3 ± 1.2 MPa, respectively. The tangent moduli of ONs and ON sheaths at 3% strains were 0.90 ± 0.56 MPa and 1.28 ± 0.50 MPa, respectively. At 6% strain, tangent moduli of anterior, equatorial, posterior, and peripapillary sclera were 22.6 ± 6.6 MPa, 22.2 ± 5.4 MPa, 10.2 ± 4.8 MPa, and 3.2 ± 1.5 MPa, respectively. The tangent moduli at 7% strain of the ON and ON sheath were 1.39 ± 0.61 MPa and 3.02 ± 1.31 MPa, respectively.

Stress σ increased exponentially with strain ε in the form σ~ε^γ^, where γ is a variable exponent. Due to the significant influence of γ, we transformed the data into log–log format: log(σ) = γlog(ε). A single γ value suited all tissues except the ON sheath, which displayed an inflection in the stress–strain curve above which slope increased. Bilinear curve fitting was applied to identify the transition point, achieving a 0.953 goodness of fit in the low strain region and 0.991 in the high strain region. The transition was at around 3.4% strain (Figure 8b, lower right).

### 3.4. Finite Element Modeling

We considered the effects of strain stiffening in the ON sheath (Figure 8), for the case of 5% pre-stretch to the ON and its sheath at the initial position of 26° adduction. The FEM then simulated the effects of incremental adduction from 26° to 32°, with or without this pre-stretch. A total of 11 eye models were simulated, corresponding to the eyes tested by 3D-DIC.

**Figure 9:**
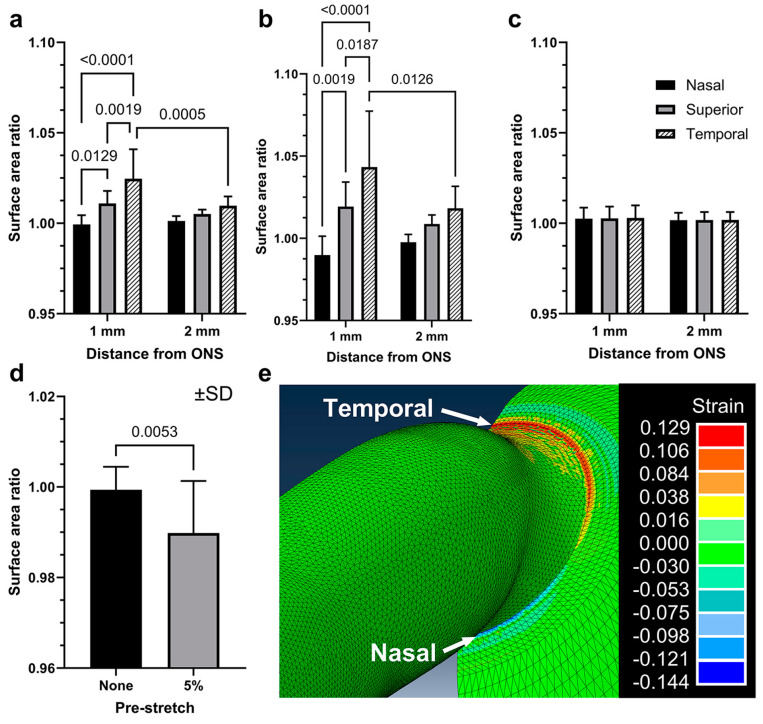
Surface area ratios from eye-specific FEMs. (**a**) Adduction without 5% pre-stretch. (**b**) Adduction with 5% pre-stretch. (**c**) IOP elevation from 15 to 30 mmHg. (**d**) Adduction loading was predicted to compress the surface area ratios in the nasal region within 1 mm of the ON sheath, but was significant only with pre-stretch of the ON and ON sheath. (**e**) Visualization of largest modulus principal strains for adduction with 5% pre-stretch for the eye in the first row in Table 1. SD—standard deviation. Paired t-tests and two-way ANOVA.

The sclera was divided into six analytic regions for comparison of DIC data with FEMs (Figure 7a). With or without 5% pre-stretch, temporal scleras exhibited significant strain during incremental adduction from 26° to 32°. However, nasal scleras exhibited significant compression only in models incorporating the 5% pre-stretch, but not without pre-stretch (Figure 9d, paired t-test, *p* = 0.0053). Deformations of nasal scleras in FEMs with 5% pre-stretch were confined to within 1 mm of the ON sheath insertion, and they were not significant 2 mm from it. For IOP elevation from 15 to 30 mmHg without adduction, FEMs predicted no significant changes from unity in surface area ratios in any scleral regions (Figure 9c). For comparison, simulated largest modulus principal strains are demonstrated for a 5% pre-stretched adduction FEM of an eye from an 82-year-old Caucasian man (Figure 9e) whose ocular dimensions are specified in the first row of Table 1.

The correlation between surface area ratio changes and other variables was investigated using GEE models employing as covariates donor age, axial globe length, transverse globe diameter, and tangent moduli of all tissue regions at 3% and 7%. None of these covariates was significantly associated with changes in surface area during adduction (*p* > 0.216). However, the ON sheath radius was negatively correlated with the surface area changes (*p* = 0.046).

**Figure 10:**
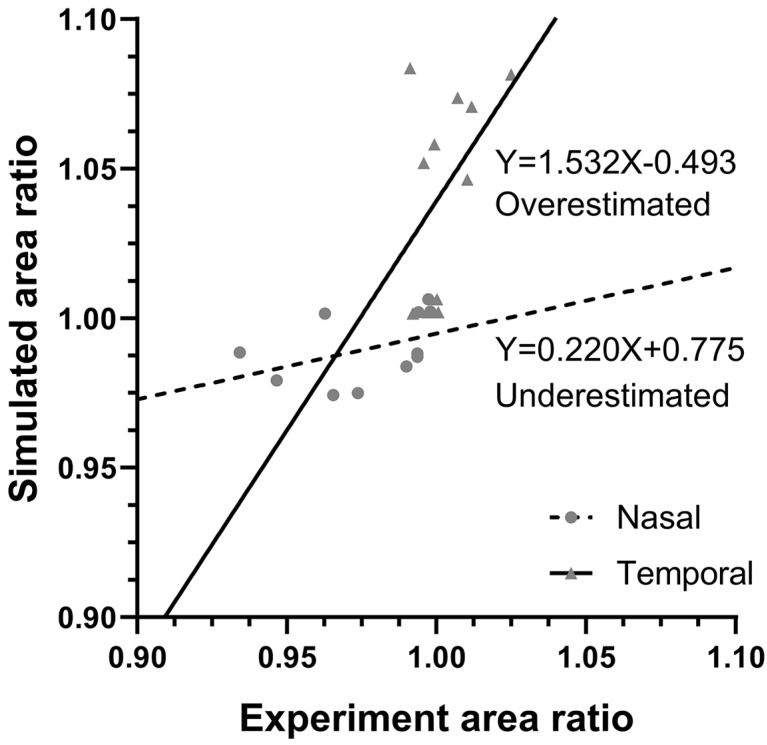
Correlation of observed changes, and changes predicted by individualized FEMs, in posterior scleral surface area caused by incremental adduction from 26° to 32° (11 eyes). FEMs incorporated 5% pre-stretch in the ON and ON sheath. Each symbol represents a nasal or temporal scleral region, 1 mm from the ON sheath border. Solid and dotted lines represent linear regressions.

Surface area ratios during adduction from 26° to 32° observed by DIC for individual eyes were compared by linear regression with predictions of eye-specific FEMs for scleras nasal and temporal to the ON sheath (Figure 10). For the nasal scleral region, simulations overestimated low and underestimated higher compression; the slope of the regression of simulated vs. observed area change was subunit at 0.220 with a 0.181 coefficient of determination. In temporal regions, simulations overestimated deformations, with a slope of 1.53 and a 0.204 coefficient of determination.

## 4. Discussion

This ex vivo study replicated a protocol that has been performed in living people using MRI to examine effects of ON tethering caused by incremental adduction from 26–32° [23,24]. While the MRI studies have demonstrated that the ON becomes straight and elongated during this adduction, the resulting local scleral deformations were not detectable due to limited MRI resolution. The focus of the current study was on the sclera observed from a posterior perspective. Adduction significantly compressed the sclera adjacent to the nasal side of its junction with the ON sheath. Individualized FEMs assembled for each eye using tensile properties measured in the ON, ON sheath, and local regions of the sclera for that eye predicted observed changes in the regional scleral surface area.

Comparison between adduction tethering and IOP elevation requires a nuanced approach. Adduction tethering generated larger deformations of the posterior scleral surface than IOP elevation, but this observational perspective is unusual insofar as most other studies of IOP elevation have focused on internal observations of the disc and peripapillary tissues [20]. Even so, this study highlighted that the main driver of the intraocular peripapillary deformations was traction by the ON and its sheath. Given the mechanical stiffness of the peripapillary sclera and anatomical continuity with the optic disc and lamina cribrosa, it is plausible that posterior scleral deformation caused by adduction could be transmitted to the adjacent lamina cribrosa with a magnitude comparable to or exceeding those due to IOP elevation. The current findings are consistent with reports that deformations of the lamina cribrosa by adduction are comparable to those due to IOP elevation [16,18,37]. For example, an OCT study in 228 subjects demonstrated that strains in the lamina cribrosa during only 20° adduction are in the range of those during IOP elevation from 16 to 35 mmHg [37]. Therefore, this current study supports the previous reports that highlight the biomechanical impacts of ON traction during eye movements.

Some previous reports exist on ocular tensile properties [19,33,38,39,40,41,42]. In addition to individual variability among donors of various ages, tensile properties of ocular tissues may depend on experimental conditions such as strain rates [43] and stress levels [38]. Although reports vary for human sclera [33,38,39,40,42], uniaxial tensile tangent moduli at low strains are usually around 10 MPa [38,42] but exceed 20 MPa for higher strains [33,38,40,42]. For human peripapillary scleras, reported tensile tangent moduli have been reported to be less than 10 MPa [33,41]. The mechanical properties of the peripapillary sclera estimated by inverse FEMs [20,41] are within the order of magnitude reported here. The tangent modulus for the human peripapillary sclera has been reported to be 8.6 MPa at 7% strain [33], higher than in the current study. The peripapillary sclera was tested in a 7.5 mm in diameter annular region in the current study, unavoidably limiting the specimen aspect ratio to about 2, although the aspect ratio would preferably exceed 5 for uniaxial tensile testing [44]. Direct tensile measurements of the human lamina cribrosa are scarce and difficult to obtain due to its small anatomical size. Due to the anatomical proximity and similar histological features of the lamina cribrosa and anterior optic nerve, the tensile modulus of the lamina cribrosa was compared to the measured modulus of the ON. The lamina cribrosa has been elsewhere estimated to have a modulus of 0.3 MPa [19], which is comparable to the 0.9 MPa value at 3% strain measured for the ON in the current study. For the human ON sheath, the previously reported tangent modulus at 7% was 11.5 MPa, with a 0.05 N preload [33]. The preload in the current study was 0.02 N, so the preload might account for some difference in observed toe region behavior.

It has been suggested from OCT in living humans that 26° adduction constitutes a critical angle beyond which deformation of peripapillary tissues increases significantly [25]. This angle has been interpreted to be the threshold at which the sinuous ON exhausts its redundancy and becomes straight [18]. Tensions in the ON and its sheath beyond 26° are influenced by stress–strain curves. The current tensile tests show that ON sheath stiffness increases markedly beyond 3.4% strain. Assuming a spherical eye of 12 mm radius [45] in an orbit having average dimensions [18], the distance between the orbital apex and ON junction lengthens by 6.6% at 26° adduction during average measured globe translation [46]. Considering 3% sinuosity of the ON in central gaze [23,24], strain in the ON and its sheath reaches 3.6% at 26° adduction, close to the stress–strain curve transition point illustrated in the lower right panel of Figure 8. This suggests that strain stiffening occurs within the physiological range of adduction, providing an alternative interpretation to the notion that ON straightening at the 26° threshold is the cause of markedly increased deformation of the optic disc and peripapillary tissues beyond this angle [25]. Such logic justifies the inclusion of pre-stretch in FEMs of adduction.

This study implies a potentially pathological significance of adduction tethering for the optic disc and peripapillary region. Scleral deformations caused by ON and sheath traction in adduction are concentrated focally at the ON junction and propagate at most 1–2 mm from the edge of the sheath on the exterior scleral surface. However, at the external surface, the ON sheath radius is much larger at 2.85–3.65 mm (Table 1) than the roughly 0.75 mm radius of the optic disc internal to the eye. In fact, deformations of the disc, peripapillary vessels, and choroid observed intraocularly by cSLO [8,9] and OCT [3,4,5] during horizontal duction occur largely within the region covered on the exterior surface by the ON sheath, and so are invisible to the current external imaging technique. This internal region, which includes the lamina cribrosa, is the site of ON damage in POAG, the world’s largest cause of untreatable blindness [47,48]. Although IOP has been believed to be the cause of POAG, high IOP is no longer definitional of the disease. Many patients, especially Asians [49,50,51,52,53], do not have abnormally high IOP [54], and experience ON damage when IOP is normal [55], as for 30–39% of white [56,57,58], 57% of Black [59], 70% of Chinese [53], and 92% of Japanese [50] people. However, treatment to reduce IOP does not always arrest optic nerve damage [60], so about 20% of patients suffer more damage 5 years after 30% IOP reduction from normal [58], even when IOP is reduced so much that its insufficiency damages sight [61]. Other causes of glaucoma besides IOP have been proposed to include the pressure gradient across the lamina cribrosa of IOP against intracranial pressure [62,63,64,65] or abnormal blood flow in the disc or ON [66], yet the etiology of POAG remains uncertain [55]. Some studies have found no strong relationship between indirectly estimated translaminar pressure and glaucoma [67], as well as the absence of a relationship when gravitational posture was varied during direct measurement of translaminar pressure [68].

Eye movement-related deformation has been proposed as another mechanical etiology for POAG [2,16,17,18,22]. While the healthy ON stretches during adduction tethering, in POAG the ON fails to stretch, abnormally retracts the globe [24], and exaggerates strain on the disc [37]. Deformations of the disc and Bruch’s membrane produced by eye movements exceed IOP-related deformations suggested as pathological to the retina [69], and many-fold those resulting from extreme IOP elevation [70]. Based on FEMs, Wang et al. predicted that 13° abduction induces larger strains of the ON head than does an IOP of 50 mmHg [15]. Consistent with the current findings, the OCT study of Chuangsuwanich et al. reported that adduction induced larger ON head strains for patients with normal-tension glaucoma than for glaucoma patients with elevated IOP [37]. It has been proposed that deformations of the scleral flange due to eye movement might lead to choroidal degeneration observed in glaucoma [71]. If eye movements do drive ON pathology in POAG, therapeutic alternatives to medical and surgical IOP reduction might eventually be considered.

Several limitations of the current study should be acknowledged. The FEMs assumed tissues to be isotropic. This could influence findings because many fibers in the peripapillary sclera are circumferentially oriented [72,73]. Biaxial tensile testing was not practical to perform in this study; while biaxial tensile data might have improved the accuracy of the simulations somewhat, it would probably not have had a large effect since the posterior sclera has been reported to behave isotropically [74]. We have previously reported roughly isotropic behavior of the human optic nerve sheath when tested without preconditioning [75], but have recently recognized that anisotropy emerges after preconditioning and that anisotropy may influence the effect of adduction tethering in the optic disc and peripapillary sclera [76].

Due to imaging path occlusion by the ON gripper, it was not possible to position a camera to capture the sclera inferior to its junction with the ON. More sophisticated mechanical characterizations that consider tissue anisotropy could improve the accuracy of the FEMs. While scleral thickness might influence FEMs, they were not adjusted for variation in scleral thickness, or deviations from spherical globe shape. Prestress over the tissues was not considered in the FEM, although its inclusion enhances simulation accuracy [77]. The mechanical system for this study considered numerous anatomical details while permitting optical imaging from a posterior perspective, but could not incorporate a realistic connective tissue suspension for the eye, and consequently prohibited globe translation. Fortunately, translation was probably not a significant factor since measured globe translation in normal subjects is close to zero during adduction from 26° to 32° [46]. Orbital fat was omitted from the experimental system, potentially resulting in overestimated scleral deformations. However, since the shear modulus of orbital fat is low [78], omission of fat would likely have little effect on the posterior sclera. Suturing of the eye to a rigid holder would alter forces on the sclera anteriorly, but have minimal effects near the ON junction where DIC was performed. Postmortem changes of the ocular tissues might influence biomechanical behavior; the tissues studied here were probably as fresh as practically achievable from human donors, and were obtained within the 72 h window within which scleral viscoelastic properties have been shown to remain stable [29]. 

## Figures and Tables

**Table 1:** Specimens tested.

No.	Axial Length (mm)	Transverse Diameter (mm)	ON Length (mm)	ON Sheath Outer Radius (mm)	Enucleation to Delivery Time (h)
1	23.7	23.2	14.4	3.65	38
2	25.9	24.3	22.0	3.12	28
3	25.7	24.5	17.2	2.85	28
4	24.2	23.9	15.1	3.05	65
5	24.5	24.2	13.4	3.12	65
6	24.5	23.1	19.9	3.25	31
7	24.5	23.0	18.1	2.81	31
8	25.0	25.6	23.5	3.21	31
9	25.1	24.7	16.0	3.03	42
10	25.9	25.9	17.4	2.86	32
11	25.8	25.8	17.7	3.51	32

ON—optic nerve.

**Table 2:** Surface area ratio mean absolute difference from unity.

	IOP Elevation 15 to 30 mmHg	Adduction 26° to 32°
1 mm nasal	0.0057	0.0228
1 mm superior	0.0019	0.0062
1 mm temporal	0.0062	0.0072
2 mm nasal	0.0032	0.0071
2 mm superior	0.0019	0.0033
2 mm temporal	0.0061	0.0064

## Data Availability

FEM files are deposited in Zenodo, 10.5281/zenodo.10622409. Image data are deposited in Zenodo, 10.5281/zenodo.10622429. Tensile stress–strain raw data are deposited in Zenodo, 10.5281/zenodo.10622442.
